# Domestic violence against women during COVID-19 pandemic restrictions

**DOI:** 10.1192/j.eurpsy.2021.167

**Published:** 2021-08-13

**Authors:** R. Keynejad

**Affiliations:** Health Service And Population Research; Section For Womens Mental Health, Institute of Psychiatry, Psychology and Neuroscience, London, United Kingdom

**Keywords:** Intimate partner violence, Gender-based violence, domestic violence, COVID-19

## Abstract

**Introduction:**

In the United Kingdom(1) and internationally(2), help-seeking for domestic violence (DV) and domestic homicides have increased(3) during COVID-19 lockdown periods. Suspension and remote delivery of face-to-face clinical services, continuing healthcare and other support services limits opportunities for DV detection and disclosure.

**Methods:**

This presentation will summarise changes in DV incidence and help-seeking during COVID-19, their impacts on health and wellbeing, and present guidance for clinicians assessing and supporting survivors.

**Results:**

World Health Organisation recommendations to Listen, Inquire, Validate, Enhance safety and Support (’LIVES’) survivors of DV remain the cornerstone of first-line support (4). Urgently-issued guidelines on safeguarding(5) and responding to DV during COVID-19(6) make a range of recommendations for clinicians supporting people experiencing DV.

**Conclusions:**

DV is an important social determinant of physical and mental health, with a range of potential fatal and non-fatal consequences. Despite the constraints of healthcare during a pandemic, attention to patients’ risk of DV and its consequences is a crucial part of bio-psycho-social assessment and management planning. References: (1) Kelly, Morgan. Coronavirus: Domestic abuse calls up 25% since lockdown, charity says. 2020. https://www.bbc.co.uk/news/uk-52157620 (2) Graham-Harrison, et al. Lockdowns around the world bring rise in domestic violence. 2020. https://www.theguardian.com/society/2020/mar/28/lockdowns-world-rise-domestic-violence (3) Roesch, et al. Violence against women during covid-19 pandemic restrictions. BMJ 2020;369. (4) WHO. Responding to intimate partner violence and sexual violence against women: WHO clinical and policy guidelines. 2013. https://apps.who.int/iris/bitstream/handle/10665/85240/9789241548595_eng.pdf;jsessionid=E19DCC3CDAB9BE390EE6F8360C6F1D7E?sequence=1 (5) RCGP. COVID-19 and Safeguarding. 2020. https://elearning.rcgp.org.uk/pluginfile.php/149180/mod_resource/content/2/COVID-19%20and%20Safeguarding%20%286%29.pdf (6) IRISi. Guidance for General Practice teams responding to domestic abuse during telephone and video consultations. 2020. https://irisi.org/wp-content/uploads/2020/04/Guidance-for-General-Practice-Covid-19-FINAL.pdf

**Disclosure:**

No significant relationships.

